# Progress in the Application of Carbon Dots-Based Nanozymes

**DOI:** 10.3389/fchem.2021.748044

**Published:** 2021-09-24

**Authors:** Jun Jin, Linlin Li, Lihui Zhang, Zhihui Luan, Shuquan Xin, Kai Song

**Affiliations:** School of Life Sciences, Changchun Normal University, Changchun, China

**Keywords:** nanozymes, carbon dots, biomedicine, catalysis, sensing, detection

## Abstract

As functional nanomaterials with simulating enzyme-like properties, nanozymes can not only overcome the inherent limitations of natural enzymes in terms of stability and preparation cost but also possess design, versatility, maneuverability, and applicability of nanomaterials. Therefore, they can be combined with other materials to form composite nanomaterials with superior performance, which has garnered considerable attention. Carbon dots (CDs) are an ideal choice for these composite materials due to their unique physical and chemical properties, such as excellent water dispersion, stable chemical inertness, high photobleaching resistance, and superior surface engineering. With the continuous emergence of various CDs-based nanozymes, it is vital to thoroughly understand their working principle, performance evaluation, and application scope. This review comprehensively discusses the recent advantages and disadvantages of CDs-based nanozymes in biomedicine, catalysis, sensing, detection aspects. It is expected to provide valuable insights into developing novel CDs-based nanozymes.

## Introduction

Natural proteases are easily denatured and degraded under harsh environmental conditions, their catalytic efficiency is limited, and their product separation and purification are costly. Their recovery and recycling are difficult, dramatically limit their practical applications (Attar et al., 2019; Wang Z. et al., 2020; Ding et al., 2020). For instance, although considerable progress has been made in the design and development of catalytic nanomotors such as bimetallic nanorods, catalytic microtubes, Janus particles and bioenzyme-driven motors, some problems remain, such as a small number of applied enzymes, a slow motor speed, and toxicity of high hydrogen oxide (H_2_O_2_) concentrations (Ma et al., 2016; Xu et al., 2019; Hermanova and Pumera, 2020; Hermanova and Pumera, 2020; Mathesh et al., 2020; Yang Q. et al., 2021; Yuan et al., 2021).

In this case, it is necessary to identify a suitable enzyme substitute to simulate the natural enzyme. Since Yan and his colleagues first demonstrated the peroxidase activity of magnetic Fe_3_O_4_ nanoparticles (NPs) in 2007, numerous nanomaterials mimicking enzymes have been developed (Gao et al., 2007; Natalio et al., 2012; Hou et al., 2013; Wei and Wang, 2013; Lin et al., 2014; Kluenker et al., 2017). In addition, the researchers are exploring ways to integrate other nanomaterials with nanozymes to improve the catalytic efficiency of cascade reactions. For example, integrated nanozyme invertase/GOx/hemin@ZIF-8A has a 700% higher catalytic efficiency than mixed invertase@ZIF-8, GOx@ZIF-8, and hemin@ZIF-8 alone (Cheng et al., 2016).

**FIGURE 1:**
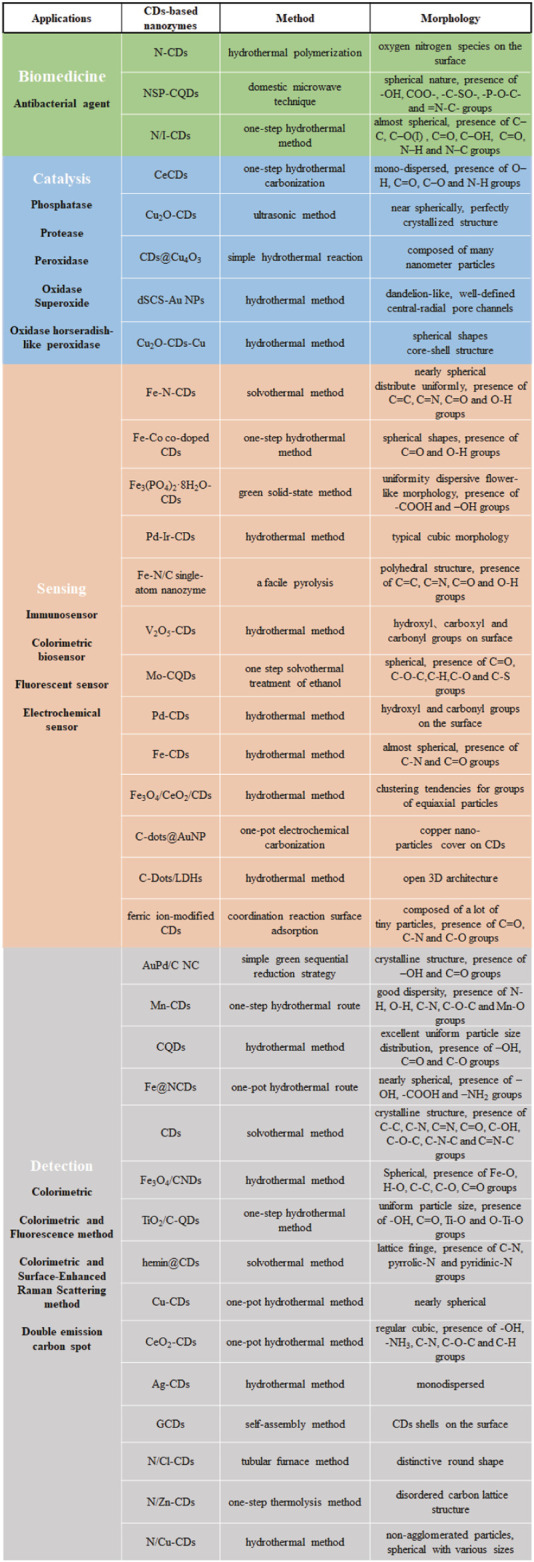
The synthesis method and structural property of CDs-based nanozymes.

CDs are excellent candidates for nanomaterial composites with nanozymes due to their surface modification, heteroatom doping, and composite with NPs (Kang and lee., 2019; Yang et al., 2020; Wang et al., 2019). In recent years, although CDs-based nanozymes have successfully simulated the structure and function of common natural enzymes such as oxidase, catalase and superoxide dismutase, they continue to face numerous obstacles (Zhao et al., 2020; Li et al., 2020a). The most significant limitation is that catalytic reactions are relatively few in number, with a strong emphasis on reduction-oxidation (REDOX) reactions. As a result, it is necessary to summarize the application research of CDs-based nanozymes with different sources and structural characteristics (Figure 1), which can provide a reference for future searching or designing novel nanozymes.

## The Applications of CDs-Based Nanozymes in Biomedicine

Biomedicine urgently requires the development of effective antimicrobial agents to combat bacterial contamination. Although antibiotics, metal NPs, composite NPs, and enzymes have been employed as antimicrobial agents, these materials exhibit several limitations: cytotoxicity, antibiotic resistance, and environmental pollution (Fischbach and Walsh, 2009; Kohanski et al., 2010; Song et al., 2012; Fasciani et al., 2014; Rizzello and Pompa, 2014; Leidinger et al., 2015). Therefore, there is a great demand for low-cost, sustainable, and effective antimicrobials suitable for long-term use. CDs-based nanozymes are an effective alternative to the above materials due to their unique electronic, optical, thermal, and mechanical properties. Zhang et al. synthesized a series of nitrogen-doped CDs to mimic the activity of oxidase. Such CDs can mimic the oxidation reaction in a few seconds and effectively inhibit the growth of *Escherichia coli* (*E. coli*) and *Salmonella* (Zhang et al., 2018). However, it demonstrated antibacterial activity only at acidic pH and insufficient activity at physiological conditions around neutral pH. For this reason, Kumud Malika Tripathi et al. prepared luminescent N, S, and P-co-doped carbon quantum dots (NSP-CQDs) that exhibited peroxidase activity over a wide pH range attributed to the presence of a high density of active sites for enzymatic-like catalysis and accelerated electron transfer during peroxidase-like reactions. It can significantly inhibit cell wall growth of *E. coli* and *Staphylococcus aureus* (Tripathi et al., 2020). Although this study realized the antibacterial effect of CDs-based nanozymes, it did not consider the toxicity issues associated with a high H_2_O_2_ concentration. Therefore, Wang et al. used a hydrothermal method to synthesize a novel nitrogen-iodine co-doped CDs (N/I-CDs) with excellent peroxidase activity. When activated by light, they catalyze the conversion of exogenous H_2_O_2_ into hydroxyl radical (OH), reduce high concentration of H_2_O_2_ to benign biological concentration (50–100 μM), and increase the cell level of reactive oxygen species (ROS) in bacterial cells. They also effectively resist Gram-negative and Gram-positive bacterial infection and accelerate the healing of artificial wounds (Wang X. et al., 2021).

At present, only a few reports are evaluating the antibacterial properties of CDs-based nanozymes. In addition, whether CDs-based nanozymes can inhibit fungi or viruses is a field worthy of research (Fan et al., 2018).

## The Applications of CDs-Based Nanozymes in Catalysis

Most catalytic reactions of nanozymes are mainly focused on peroxidase, oxidase, superoxide oxidase, and catalase reactions, while natural enzymes are diverse and exhibit various catalytic capabilities, developing nanozymes for new enzyme reactions is highly demanding (Gao et al., 2007; Asati et al., 2009; Wei and Wang, 2013; Lin et al., 2014). For instance, Ce-doped CDs (CeCDs) can simulate phosphatase activity, which is used for phosphate ester hydrolysis (Du et al., 2020). However, the optimal reaction conditions for this nanozyme are an alkaline solution with pH 8.5 and a high temperature of 200°C. These harsh reaction conditions significantly limit its application in biological systems. Li et al. attempted to synthesize Cu_2_O-decorated carbon quantum dots (Cu_2_O-CDs) with intrinsic protease-simulating activity, which hydrolyzed proteins including bovine serum albumin and casein under physiological conditions (Li et al., 2020a). This dramatically improves the applicability of nanozymes in proteomics and related fields, opening the door to a plethora of potential biological applications.

As many biochemical processes are carried out by various enzymes, studying nanozymes simulating complex enzyme reactions is one of the demanding research goals. Li et al. studied paramelaconite (CDs@Cu_4_O_3_) with both oxidase and peroxidase activities (Li et al., 2018). Zhao et al. synthesized dual nanozymes with a complex CDs, which realized the simultaneous dual catalysis of superoxide dismutase and horseradish peroxidase activities (Zhao et al., 2020). These CDs-based nanozymes provide a new perspective on synergistic properties and comprehensive functions beyond traditional nanozymes. In addition, the properties of composite materials can confer the nanozymes new properties, such as stimulus responsiveness. Li et al. synthesized Cu_2_O-CDs-Cu three component oxidase-like catalyst, which can effectively generate high-energy electrons under visible light irradiation to improve its oxidase catalytic activity (Li et al., 2020b). This study provides insights into the design of catalysts that can effectively couple thermal and photonic stimuli to drive oxidase-like activity.

The catalytic mechanism of CDs-based nanozymes is not fully understood. Although the active intermediates, catalytic activity, and substrate binding sites have been identified, the progression of reactions remains unclear.

## The Applications of CDs-Based Nanozymes in Sensing

As an ideal and essential tool of biosensors, nanozymes have attracted great attention because of their lower cost, higher stability and more convenient preparation than protein enzymes. Inorganic nanomaterials with various enzymatic activities, such as ferromagnetic NPs, AuNP@MoS_2_QD gold NPs, and MoS_2_ Nanoribbons, have been explored as biosensors (Wei and Wang, 2013; Woo et al., 2013; Nirala et al., 2015; Liu et al., 2018; Vinita et al., 2018; Ding et al., 2019).

### The Immunosensor

Yang et al. synthesized iron and nitrogen co-doped CDs (Fe-N-CDs), which with peroxidase activity. 3,3′,5,5′-tetramethylbenzidine (TMB) was catalyzed to blue in the presence of hydrogen peroxide. On this basis, Fe-N-CDs conjugated antibody was applied to detect carcinoembryonic antigen (CEA) by immunosorbent assay. The detection limit was as low as 0.1 p g/mL within 5 min (Yang et al., 2017). Based on the similar principle of enzyme-linked immunosorbent assay, iron and cobalt co-doped CDs with high peroxidase-like activity and palladium-iridium nanocubes with CDs as reference fluorophores can detect histamine and cardiac troponin I, respectively (Tan et al., 2019; Li et al., 2021). Even more striking, Guo et al. used Fe_3_(PO_4_)_2_·8H_2_O-CDs-FA hybrid nanoflower realized the naked eye immunoassay of as few as 25 HeLa cells (Guo et al., 2019).

### The Colorimetric Biosensor

Based on the above TMB discoloration principle, Fe-N/C single-atom nanozyme was used to screen alkaline phosphatase activity in the range of 0.05–100 U/L, with a detection limit of 0.02 U/L (Chen Q. et al., 2020). The cascade colorimetric biosensor combined with cholesterol oxidase demonstrated excellent selectivity and high sensitivity to the target in the concentration range of 0.01–1.0 mM. The detection limit was as low as 7 mM (Zhao et al., 2019). Both V_2_O_5_-CDs nanocomposites and palladium/CDs composites (Pd-CDs) have also been proved to bind glucose oxidase and realize the colorimetric glucose sensing with a detection limit as low as 0.2 μM (Honarasa et al., 2019).

### The Fluorescent Sensors

CDs have demonstrated significant application value in fluorescence detection due to their numerous unique physical and photochemical properties, and CDs-based nanozymes also exhibit fluorescence detection characteristics (Zhan et al., 2020).

Lu et al. synthesized Fe-doped CDs (Fe-CDs). Oxidative OPD (ox-OPD) can be generated when the oxidase substrate o-phenylenediamine (OPD) coexists with H_2_O_2_. Therefore, a dual fluorescence emission detection system can be established based on fluorescence characteristics of Fe-CDs and Ox-OPD. The results indicated that the limit of detection for cysteine was as low as 0.047 μM in the concentration range of 0.25–90 μM (Lu et al., 2020).

### The Electrochemical Sensors

The advantages of electrochemical sensors include linear output, low power consumption, good resolution, repeatability, and accuracy (Chen et al., 2019; Teymourian et al., 2020; Wang Q. et al., 2021). Additionally, applying CDs-based nanozymes to electrochemical sensors is a hot topic.

The realization of electrochemical sensing based on CDs-based nanozymes is often the modification of electrodes. Wang et al. immobilized horseradish peroxidase on a glassy carbon electrode by simply mixing carbon nanodots and cobalt-iron layered double hydroxides (Wang et al., 2015). Qin et al. used hydroxyl-rich carbon dot-assisted gold nanoparticles (CDs @AuNP) as a marker of copper deposition reaction, and cooperated with chitosan to modify glassy carbon electrode (Qin et al., 2018). Hu et al. used coordination reaction and surface adsorption to prepare ferrous and ferrous ion modified CDs to regulate heterogeneous nucleation process of iron oxide, and its enzyme-like activity was more than 6 times higher than that of pure Fe_2_O_3_ nanomaterials (Hu et al., 2021) Fatemeh Honarasa et al. prepared Fe_3_O_4_/CeO_2_/C-dot nanozyme with more complex structure, and its modified multi-walled carbon nanotube/ionic liquid paste (MWIL) electrode was used for electrocatalytic determination of H_2_O_2_, showing a linear range of 2.0 × 10^−8^ ∼ 1.0 × 10^−6^ M (Honarasa et al., 2021).

Compared with metal/metal oxide NPs or materials, CDs have the disadvantages of low product yield, difficulties in purification and precise size control, which significantly affect applying CDs-based nanozymes in biosensors.

## The Applications of CDs-Based Nanozymes in Detection

### The Colorimetric Detection

Biomolecules such as H_2_O_2_, ascorbic acid, uric acid, and pyrophosphate have also been developed to detect the nanozyme complex CDs method.

Yang et al. synthesized carbon-based AuPd bimetallic nanocomposite (AuPd/C NC) with good catalytic activity and peroxidase activity. H_2_O_2_ can be detected in a wide linear concentration range of 5–500 µM and 500 µM–4 mM (Yang et al., 2016). Zhuo et al. demonstrated that manganese (II) doped CDs (Mn-CDs) have a similar catalytic ability to oxidase. They could be utilized for quantifying ascorbic acid in a concentration range of 50–2,500 nM based on the principle of “TMB discoloration reaction” (Zhuo et al., 2019). Shu et al. demonstrated that carbon quantum dots (CQDs) also exhibit peroxidase activity but with a narrower detection range and lower detection limit (Shu et al., 2020). Liang et al. synthesized carbon quantum dots co-doped with iron and nitrogen (Fe@NCDs). In the presence of H_2_O_2_, the response was linear in the uric acid concentration range of 2–150 μM (Liang et al., 2020). Chen et al. prepared nanozymes with complex CDs exhibiting peroxidase simulation properties, which could catalyze o-phenylenediamine oxidation in the presence of H_2_O_2_. The process was inhibited by pyrophosphate (PPI), and the degree to which it was inhibited could be monitored using the colorimetric method with generated yellow product 2,3-diaminophenazine (Chen Q. et al., 2020).

Although nanozyme-based colorimetry is a rapid method for detecting glutathione, it lacks the high efficiency and low toxicity of nanozyme. Luo et al. prepared Fe_3_O_4_/CNDs hybrid NPs with excellent peroxidase-like catalytic activity, and they could produce a rapid color reaction on glutathione (Luo et al., 2019). Similar studies have focused on peroxidase-like nanomaterials, which require H_2_O_2_ addition. Because H_2_O_2_ is extremely unstable, quickly decomposes, and even reacts with assay, applying this nanozyme mimicking peroxidase remains limited. Therefore, Jin et al. prepared titanium dioxide/carbon point oxidase nanozyme. The nanozyme possessed abundant thermodynamic metastable Ti atoms on MXene. The oxygen vacancy in TiO_2_ on carbon matrix surface can facilitate O_2_ adsorption in solution, generating ROS, thereby quickly oxidizing TMB to TMBox in the absence of H_2_O_2_ to detect glutathione (Jin et al., 2020).

### Collaborative Detection by Colorimetric Method and Fluorescence Method

Although colorimetric and fluorescence methods possess high selectivity, high sensitivity, low cost, and simplicity, such methods follow single-mode signal readout. It is easy to be disturbed by the environment and challenging to meet accurate bioassay requirements. In this case, colorimetric/fluorescence two-channel measurement provides a more reliable strategy for detecting H_2_O_2_ and related biomolecules.

Su et al. prepared for the first time a multifunctional hemin@CDs hybrid nanozymes (hemin@CDs) with peroxidase-like activity and fluorescence signal properties (Su et al., 2020). This is a two-channel fluorescent probe for H_2_O_2_ and H_2_O_2_-based biocatalytic systems. It catalyzes the oxidative coupling of 4-aminoantipyrine and phenol in the presence of H_2_O_2_, resulting in a pink quinone imine dye with a maximum absorbance at 505 nm. The probe can be deployed to detect glucose and xanthine due to the conversion of glucose/xanthine into H_2_O_2_ catalyzed by related oxidase.

Ren et al. synthesized active copper-containing CDs (Cu-CDs) with inherent laccase-like activity. Unlike Su et al.‘s work, this is a novel enzyme reaction that catalyzes phenylenediamine oxidation by laccase substrates, resulting in a typical color change from colorless to brown. Cu-CDs were further employed as a fluorescent probe for unlabeled hydroquinone (H_2_Q) detection. The results indicate that a linear relationship is good in buffers with different pH values of 0.05–20 mM and 1–30 mM (Ren et al., 2015).

To further overcome the problem of obtaining fluorescence utterly dependent on a single signal output and a low signal background ratio in the method mentioned above, Yang et al. prepared CDs-doped CeO_2_ (CeO_2_-CDs) with peroxidase activity and fluorescent carbon dot. Fluorescent o-phenylenediamine (OPD), a peroxidase substrate, can be catalyzed by cerium oxide and cadmium sulfide to produce fluorescent o-phenylenediamine (palladium oxides). UV-Vis absorption of palladium oxides partially overlays the fluorescence emission of cadmium sulfide, reducing its intensity under the effect of an internal filter (Yang Z. et al., 2021). Based on this principle, a sensitive and selective fluorescence assay for the ratio of H_2_O_2_ to cholesterol was developed.

### Collaborative Detection by Colorimetric Method and Surface-Enhanced Raman Scattering (SERS) Method

Gold and silver are typical SERS substrates. The SERS activity of precious metals/CDs nanocomposites was enhanced by improving probe molecule adsorption and amplifying electromagnetic fields.

Wang et al. prepared silver-CDs (Ag-CDs) nanocomposites with excellent peroxidase and SERS activity. The nanocomposite can be used to determine uric acid (UA) levels (Wang et al., 2019). In addition, the chain-like Au/CDs (GCDs) nanocomposite was simulated using finite-difference time-domain (FDTD) method to demonstrate how the aggregation of gold NPs enhanced the electromagnetic field, thereby increasing SERS signal based on diamond-like nanocomposite. The nanocomposite enables glucose detection at a concentration of 5 × 10^−7^ M (Gan et al., 2021). All these demonstrated that the synergistic method based on colorimetric reaction and SERS detection possessed the advantages of a low detection limit, a wide detection range, and high accuracy, which made the detection results more reliable and accurate.

### Double Emission Carbon Spot Detection

Using a two-carbon point system as a peroxide-mimicking enzyme and a fluorescent probe, combining carbon point with catalytic activity or carbon point with fluorescence quenching effect greatly improves the sensitivity of the detection method.

Dhamodiran Mathivanan et al. synthesized double emission carbon spots of enzyme simulated N/Cl-CDs and N/Zn-CDs. N/Cl-CDs exhibited apparent intrinsic peroxidase-like activity, catalyzing OPD oxidation by H_2_O_2_ to form the yellow product 2, 3-diaminophenazine. N/Zn-CDs exhibited significant fluorescence properties, with a quantum yield of 27.52% (Mathivanan et al., 2020). Using similar construction, Wang et al. constructed a double-carbon point system with fluorescent CDs (N/Cl-CDs) and copper-doped CDs (N/Cu-CDs) that function as peroxide mimic and fluorescent probe and can fluoresce in hydroquinone determination. The fluorescence quantum yield of N/Cu-CDs was 37%. Compared with the study of Dhamodiran Mathivanan et al., the fluorescence quantum yield was significantly improved. N/Cl-CDs exhibits inherent peroxidase-like activity and catalyzes hydroquinone oxidation to p-benzoquinone and intermediates to determine H_2_Q (Wang X. et al., 2020).

Although nanozymes with complex CDs have the advantages of rapid response, high sensitivity, and simplicity when applied to molecular detection, they possess some limitations and are unsuitable for *in vivo* and continuous analyses. However, they lay the foundation for enzyme-dependent biological research. In future studies, it is necessary to enhance the substrate specificity of CDs complex nanozymes by modifying their functional groups.

To clearly describe the application performance of CDs-based nanozymes in the field of detection, we summarized the existing reports in Table 1.

**TABLE 1:** Summary of the application of CDs-based nanozymes in detection.

Detection method	Sample	Linear range	Detection limit	References
Colorimetric Detection	H_2_O_2_	5–500 µM	0.16 μM	Yang et al. (2016)
		500 µM-4 mM		
–	Glutathione	0.058 μM	–	Luo et al. (2019)
–	–	0.5–25 μM	0.2 μM	Jin et al. (2020)
–	Ascorbic acid	50–2500 nM	9 nM	Zhuo et al. (2019)
–	–	1.0–105 μM	0.14 μM	Shu et al. (2020)
–	Uric acid	2–150 μM	0.64 μM	Liang et al. (2020)
–	Pyrophosphate	–	4.29 nM	Chen et al. (2020b)
	Ion			
Collaborative detection by colorimetric and fluorescence methods	H_2_O_2_	–	0.11 µM (colorimetric method)	Su et al. (2020)
			0.15 μM (fluorescence method)	
–	–	1.67 µM-2.01 mM	0.35 µM	Yang et al. (2021b)
–	Glucose	–	0.15 μM (colorimetric method fluorescence method)	Su et al. (2020)
–	Xanthine	–	0.11 μM (colorimetric method)	Su et al. (2020)
			0.12 μM (fluorescence method)	
–	Hydroquinone (H_2_Q)	0.05–20 mM	1 μM	Ren et al. (2015)
		1–30 mM		
–	Cholesterol	1.66 µM-1.65 mM	0.49 µM	Yang et al. (2021b)
Collaborative detection by colorimetric and SERS methods	Uric acid	–	1–500 μM (colorimetric method)	Wang et al. (2019)
			0.01–500 μM	
			(SERS method)	
–	Glucose	–	5 × 10^–7^ M	Gan et al. (2021)
Double emission carbon spot detection	O-phenylenediamine	–	0.58 μM	Mathivanan et al. (2020)
–	H_2_O_2_	–	0.27 μM	Mathivanan et al. (2020)
–	Hydroquinone (H_2_Q)	1.0–75 μM	0.04 μM	Wang et al. (2019)

## Disscussion

In the past 10 years, CDs-based nanozymes have progressed in expanding the types of nanozymes, understanding the reaction mechanism, and regulating their catalytic performance, but numerous problems remain.

1) There is limited information on the biological characteristics of CDs-based nanozymes *in vivo.* The biological effects of CDs-based nanozymes should be systematically described, including their cytotoxicity, *in vivo* properties, biological distribution, and pharmacokinetics to facilitate their broad applications in cancer treatment, ROS removal, and inflammation alleviation.
2) The detailed system mechanism of CDs-based nanozymes remains unclear, and the relationship between the catalytic mechanism and its structure requires further investigation. By studying their structures, it is feasible to integrate enzyme-like activities and catalytic mechanisms of various nanozymes. In addition, a well-defined coordination structure can provide a clear experimental model for studying the underlying mechanism, and computational simulation can better design nanozymes with CDs.
3) To date, most CDs-based nanozymes exhibit only oxidoreductase-like activity. Given the numerous enzyme-catalyzed biochemical reactions in nature, it is necessary to further develop novel CDs-based nanozymes with a wider range of enzyme activities. In addition to stimulating proteases, it may be a breakthrough direction to broaden the simulation objects of nucleic acid-based enzymes, such as graphene oxide, as a photocatalytic nuclease, which could cleave DNA.
